# The Omega-3 Fatty Acids EPA and DHA, as a Part of a Murine High-Fat Diet, Reduced Lipid Accumulation in Brown and White Adipose Tissues

**DOI:** 10.3390/ijms20235895

**Published:** 2019-11-24

**Authors:** Nikul Soni, Alastair B. Ross, Nathalie Scheers, Intawat Nookaew, Britt G. Gabrielsson, Ann-Sofie Sandberg

**Affiliations:** 1 Division of Food and Nutrition Science, Department of Biology and Biological Engineering, Chalmers University of Technology, SE-41296 Gothenburg, Sweden; soni@chalmers.se (N.S.); alastair.ross@chalmers.se (A.B.R.); nathalie.scheers@chalmers.se (N.S.); bggabr@gmail.com (B.G.G.); 2 Food and Biobased Products Group, AgResearch Institute, 7674 Lincoln, New Zealand; 3 Department of Biomedical Informatics, College of Medicine, University of Arkansas for Medical Sciences, Little Rock, AR 72204, USA; INookaew@uams.edu

**Keywords:** eicosapentaenoic acid (EPA), docosahexaenoic acid (DHA), brown and white adipose tissue, gene expression profiling, signaling pathway, inflammation

## Abstract

Excess energy intake can trigger an uncontrolled inflammatory response, leading to systemic low-grade inflammation and metabolic disturbances that are hypothesised to contribute to cardiovascular disease and type 2 diabetes. The long chain n-3 polyunsaturated fatty acids (LC n-3 PUFAs) eicosapentaenoic acid (EPA) and docosahexaenoic acid (DHA) are suggested to mitigate this inflammatory response, but the mechanisms are unclear, especially at the tissue level. Adipose tissues, the first tissues to give an inflammatory response, may be an important target site of action for EPA and DHA. To evaluate the effects of EPA and DHA in white and brown adipose tissues, we fed male C57Bl/6J mice either a high fat diet (HFD) with 5% corn oil, an HFD with 40% of the corn oil substituted for purified EPA and DHA triglycerides (HFD-ED), or normal chow, for 8 weeks. Fatty acid profiling and transcriptomics were used to study how EPA and DHA affect retroperitoneal white and brown adipose tissues. HFD-ED fed mice showed reduced lipid accumulation and levels of the pro-inflammatory fatty acid arachidonic acid in both white and brown adipose tissues, compared with HFD-corn oil fed animals. The transcriptomic analysis showed changes in β-oxidation pathways, supporting the decreased lipid accumulation in the HFD-ED fed mice. Therefore, our data suggests that EPA and DHA supplementation of a high fat diet may be anti-inflammatory, as well as reduce lipid accumulation in adipose tissues.

## 1. Introduction

Obesity is characterized by excessive fat accumulation accompanied by weight gain, and often induces chronic low-grade inflammation, which in turn is a risk factor for type 2 diabetes (T2D), cardiovascular diseases (CVD), and other metabolic abnormalities [1]. Obesity is caused by excessive energy intake, and white adipose tissue (WAT), as the main storage organ for excess energy in the form of lipids, also activates an inflammatory response to excess energy intake [2]. The problem with obesity may be exacerbated by a decreased capability to dissipate energy through adaptive thermogenesis in brown adipose tissue (BAT) [3]. An important aspect to the role of adipose tissue in obesity is that WAT can transiently turn to beige adipocytes [4], which can use surrounding lipids through mitochondrial thermogenesis via uncoupling protein. This process is affected by gender [5] and external cues including environmental temperature [6]. Furthermore, it has recently been demonstrated that omega-3 long chain PUFA supplementation has a beneficial effect on the thermogenic function of adipocytes and compensates for the inhibitory effect of omega-6 long chain PUFA on the thermogenic function [7,8].

From a nutritional perspective, not only the quantity, but also the quality of dietary lipids may be an important factor in the development of obesity-related disease. For example, dietary lipid chain length and saturation can influence the metabolism [9,10]. A typical westernized diet often includes an excessive amount and proportion of saturated fatty acids, which increase the risk of CVD and provides too low levels of long chain n-3 polyunsaturated fatty acids (LC n-3 PUFAs) compared to n-6 PUFAs [11,12,13]. LC n-3 PUFAs are suggested to aid in preventing or ameliorating inflammatory diseases, autoimmune diseases, and consequently the risk of CVD [13,14]. Supplementation with fish oil, which is a major source of the n-3 fatty acids eicosapentaenoic acid and docosahexaenoic acid (EPA and DHA) has been suggested to prevent T2D [15], while some studies have found that marine fatty acids have no [16] or inconclusive effects on metabolic syndrome [17]. A reason for these conflicting findings may be confounding factors from other dietary components including other lipids present in fish oil, as reported in several rodent studies [18,19,20].

As adipose tissues are known to play an important role in the pathogenesis of obesity and related disorders [21,22], we hypothesised that a diet enriched with EPA and DHA would impact upon gene expression in adipose tissue which may help explain some of the findings related to human health. In order to understand this, we used transcriptomics to measure the gene expression in BAT and WAT from mice fed a high fat diet (HFD) supplemented with 5% of corn oil or the same HFD except with 40% of the corn oil substituted with EPA and DHA enriched triglycerides as a model of diet induced obesity. Furthermore, we have used EPA and DHA enriched oil, rather than fish oil, to reduce the confounding, which may come from the presence of many other compounds besides EPA and DHA in fish oil. This makes it possible to attribute the effects more specifically to these LC n-3 PUFAs. 

## 2. Results

Mice were fed with standard control chow during their acclimatization period and the mice did not differ in body weight at the start of the study (Table 1). The intervention study used three groups of mice that were fed either standard chow (control group; *n* = 11) HFD-corn oil (*n* = 12), or HFD-ED (*n* = 12). Two mice were removed from the control diet group due to fighting, hence there were n = 9 and *n* = 12 mice in control and each HFD groups, respectively. The differing HFDs differed only in their composition of fatty acids (FA) (Table 2), based on the replacement of 40 % of the corn oil with oil enriched with EPA and DHA, representing 2 % of the total diet weight and 13 % of total fat in the feed. In total, the control diet provided 24 energy% (E%) protein, 12 E% fat and 65 E% carbohydrates, whereas both the HFD provided 25 E% protein, 32 E% fat and 44 E% carbohydrates (Table 2).

### 2.1. Physiological Changes Induced by HFD-ED in Brown- and White Adipose Tissues 

**Figure 1:**
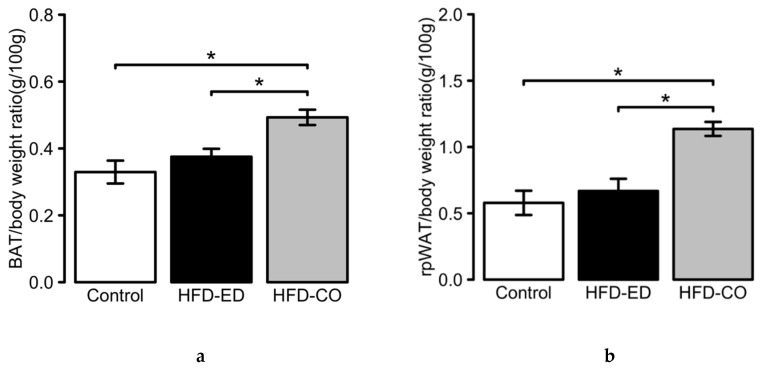
Physiological changes in adipose tissues upon dietary intervention. (**a**) Brown adipose tissue: Effects of 8 weeks of diet intervention on brown adipose tissue (BAT), represented as BAT to body weight ratio. (**b**) White adipose tissue: Effects of 8 weeks diet intervention on white adipose tissue (WAT), represented as WAT to body weight ratio. For 8 weeks dietary intervention: *n* = 10, 12, 12; control, high fat diet (HFD corn oil with 40% substituted with EPA and DHA (HFD-ED) and HFD-corn oil (HFD-CO), respectively. * asterisk denotes significant difference between evaluated groups by Tukey’s multiple comparison test. Data are shown as mean ± SEM and *p*-value < 0.05 was considered significant.

After 8 weeks of dietary intervention, we observed delayed weight gain in mice fed HFD-ED compared with HFD-corn oil (Table 1) even though the mice were fed isoenergetic diets similar in macronutrients only differing in the source of 13 % of the total fat content. Furthermore, relative tissue weight of BAT (Figure 1a) and WAT (Figure 1b) from the mice fed HFD-ED was 76 % (BAT) and 58 % (WAT) compared to feeding the mice with HFD-corn oil. No difference between the HFD-ED and control diet fed mice was observed (Figure 1). 

### 2.2. Fatty Acid Composition of the Brown and White Adipose Tissues

**Figure 2:**
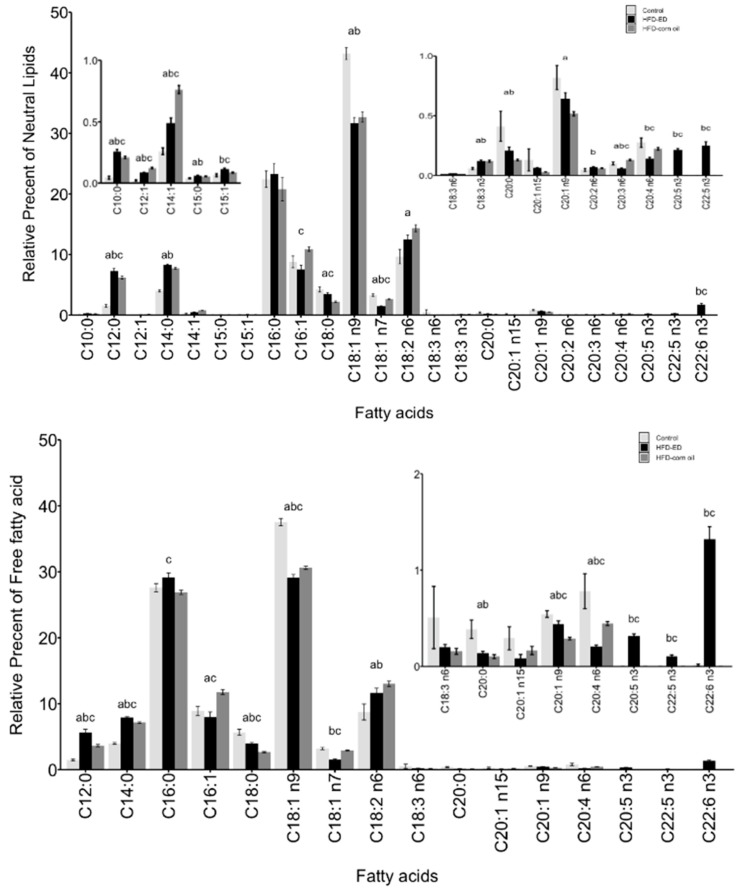

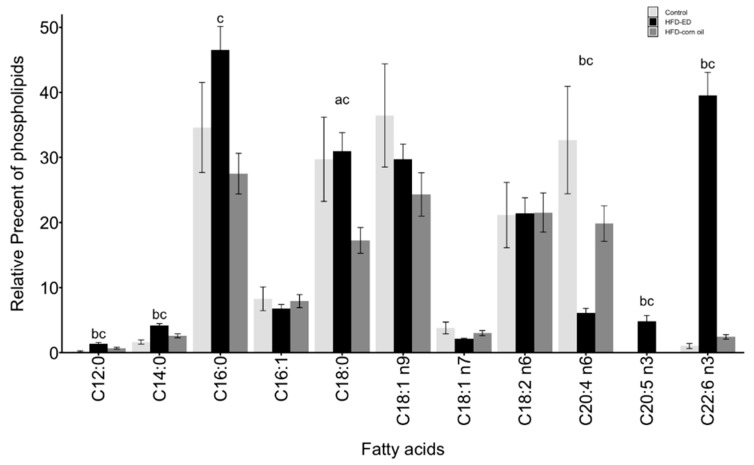
BAT.Fatty acids methyl esters (FAME) composition is shown as mean ± SEM and as a relative percentage of total lipid fractions in order of increasing number of carbon-chain length of respective fatty acid. n3 and n6 denotes omega-3 and omega-6 fatty acids, respectively. White bar control, black bar HFD-ED, grey bar HFD-corn oil (**top**) BAT neutral lipid fraction and FAME composition, (**middle**) BAT free fatty acid fraction and FAME composition, (**bottom**) BAT phospholipid fraction and FAME composition, from the mice fed either control diet (BAT *n* = 5), HFD-ED (BAT *n* = 8) or HFD-corn oil (BAT *n* = 12) after 8-week dietary intervention. The significance is denoted by different letters and the statistical difference was tested by ANOVA followed by Tukey’s multiple comparison test where letter a = *p*-value < 0.05 between HFD-corn oil vs. control diet; b = *p*-value < 0.05 between HFD-ED vs. control diet; c = *p*-value < 0.05 between HFD-ED vs. HFD-corn oil.

**Figure 3:**
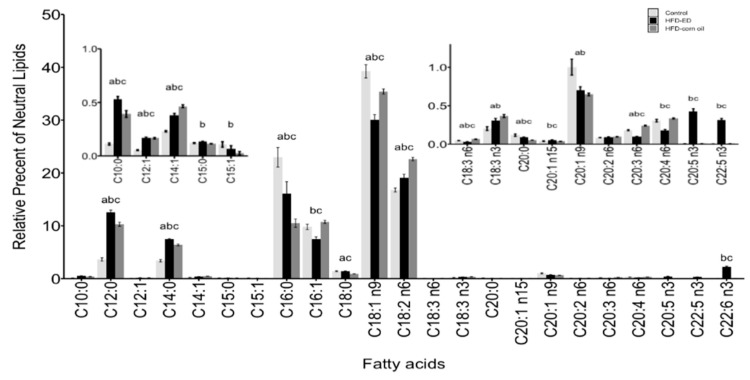

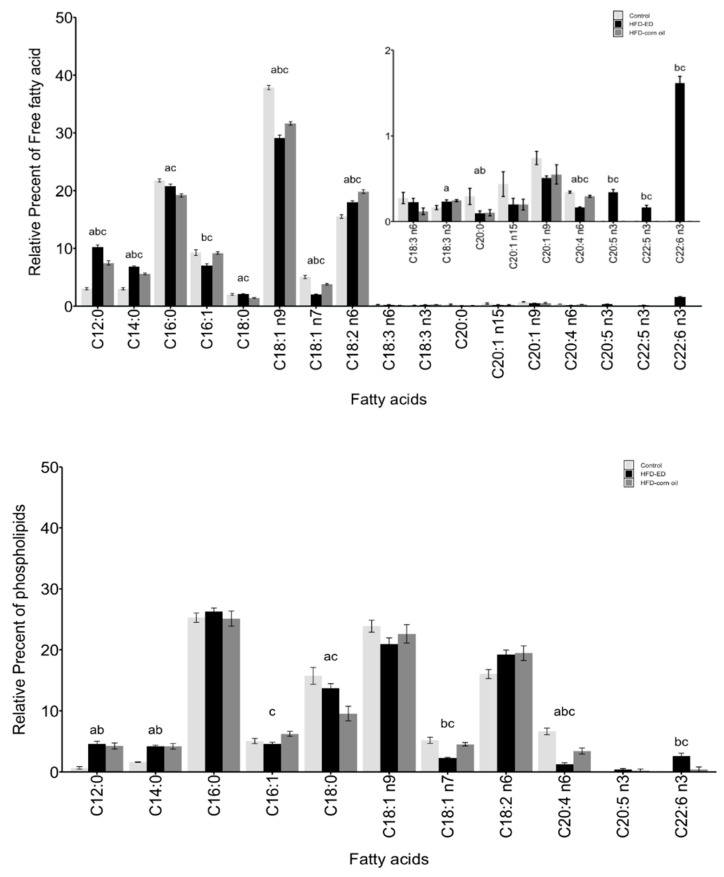
WAT.Fatty acids methyl esters (FAME) composition is shown as mean ± SEM and as a relative percentage of total lipid fractions in order of increasing number of carbon-chain length of respective fatty acid. n3 and n6 denotes omega-3 and omega-6 fatty acids, respectively. White bar control, black bar HFD-ED, grey bar HFD-corn oil (**top**) WAT neutral lipid fractions and FAME composition, (**middle**) WAT free fatty acid fraction and FAME composition, (**bottom**) WAT phospholipid fraction and FAME composition, from the mice fed either control diet (WAT *n* = 7), HFD-ED (WAT *n* = 10) or HFD-corn oil (WAT *n* = 11) after 8-week dietary intervention. The significance is denoted by different letters and the statistical difference was tested by ANOVA followed by Tukey’s multiple comparison test where letter a = *p*-value < 0.05 between HFD-corn oil vs. control diet; b = *p*-value < 0.05 between HFD-ED vs. control diet; c = *p*-value < 0.05 between HFD-ED vs. HFD-corn oil.

The neutral lipid fraction: The relative abundance of n-3 PUFAs, including C20:5 n-3 (EPA), C22:5 n-3 (docosapentaenoic acid; DPA) and C22:6 n-3 (DHA), were higher in both WAT and BAT from the mice fed HFD-ED compared with either control or HFD-corn oil (Figure 2 and Figure 3). In BAT and WAT, the relative abundance of saturated fatty acids (SFA) C12:0 and C14:0 was higher in the mice fed HFD-ED compared with either HFD-corn oil or control diet, whereas C16:0 was higher for the comparison HFD-ED *vs* HFD-corn oil only in WAT (Figure 2 and Figure 3). In WAT, the monounsaturated fatty acid (MUFA) C18:1 n-9 was lowest in HFD-ED fed mice compared with the remaining two diets (Figure 3). The relative abundance of n-6 PUFAs in BAT, including C18:2 n-6 (linoleic acid) was higher in HFD-corn oil fed compared to control diet, and C20:4 n-6 (arachidonic acid) was lower in HFD-ED compared with control and HFD-corn oil fed animals (Figure 2). The amount of n-6 PUFAs in WAT, including linoleic acid and arachidonic acid was lower in the mice fed HFD-ED compared with the mice fed either the control or HFD-corn oil (Figure 3).

Free fatty acids: In BAT and WAT, LC n-3 PUFAs including EPA, DPA, and DHA were higher in both the adipose tissues from the mice fed HFD-ED compared with control or HFD-corn oil (Figure 2 and Figure 3). Among the SFA in BAT and WAT, the relative abundance of C12:0 and C14:0 was higher in the mice fed HFD-ED compared with either HFD-corn oil or control diet, whereas the abundance of C16:0 was higher for the comparison HFD-ED vs. HFD-corn oil in BAT and in WAT (Figure 2 and Figure 3). The MUFA including C18:1 n-9 and C18:1 n7 were lowest in the HFD-ED fed mice compared with HFD-corn oil or control fed animals (Figure 2 and Figure 3). However, the relative abundance of linoleic acid and arachidonic acid was lower in the WAT from the mice fed HFD-ED compared with HFD-corn oil (Figure 3).

The phospholipid fraction: In BAT, the relative abundance of LC n-3 PUFAs including EPA and DHA was remarkable in HFD-ED compared to the control and HFD-corn oil (Figure 2). Interestingly, the amount of EPA did not differ in the WAT from the mice fed HFD-ED, but the DHA amount was higher in HFD-ED fed mice compared with the control and HFD-corn oil fed mice (Figure 3). Moreover, in BAT, the C16:0 was higher in HFD-ED fed animals compared to HFD-corn oil and the C18:0 was lower in HFD-corn oil compared with control fed animals and HFD-ED fed mice (Figure 2). The relative abundance of n-6 PUFAs in BAT, including the C20:4 n-6 (arachidonic acid) was lower in HFD-ED compared with control and HFD-corn oil fed animals (Figure 2). Also, arachidonic acid levels were lower in the WAT of the mice fed HFD-ED compared with control and HFD-corn oil (Figure 3).

### 2.3. Transcriptomic Differences between HFD-ED and HFD-Corn Oil Fed Mice.

**Figure 4:**
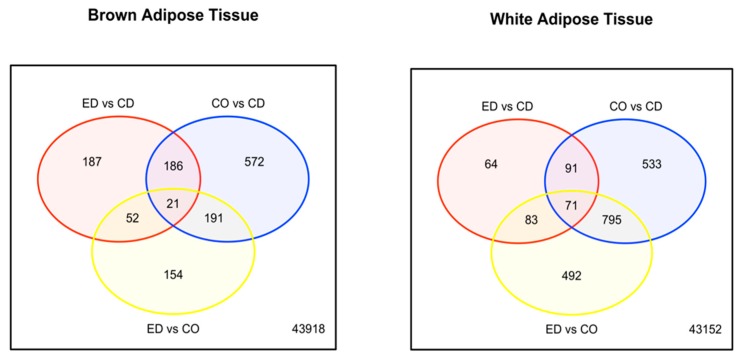
Venn diagram of differentially regulated genes at the cut off of the *p*-value < 0.001 after 8-week dietary intervention. (**Left**) Venn diagram depicting overlaps among differentially expressed genes in BAT upon dietary intervention. Most regulation can be seen for comparison HFD-corn oil vs. control diet (CO vs. CD) = blue and HFD-ED vs. HFD-corn oil (ED vs. CO) = yellow, whereas HFD-ED vs. control diet (ED vs. CD) = red shows comparatively less bidirectional differentially regulated genes. (**Right**) Venn diagram depicting overlaps among differentially expressed genes in WAT upon dietary intervention. Most regulation can be seen for comparison HFD-corn oil vs. control diet (CO vs. CD) and HFD-ED vs. HFD-corn oil (ED vs. CO), whereas HFD-ED vs. control diet (ED vs. CD) shows least bidirectional differentially regulated genes.

For the assessment of the effects of different HFD-ED and HFD-corn oil diets on BAT and WAT, differential gene-expression was analysed for the following comparisons: Comparing the two HFDs, HFD-corn oil differently regulated 1441 genes in WAT (Figure 4) and 418 genes in BAT compared to HFD-ED (Figure 4) after 8 weeks of dietary intervention. HFD-ED vs. control and HFD-corn oil vs. control were also investigated. From these comparisons, it was observed that HFD-corn oil showed most differential expressed genes (DEGs) in BAT (970 genes; Figure 4) and WAT (1490 genes; Figure 4) compared with control diet fed animals, whereas HFD-ED differed for fewer genes in BAT (446 genes; Figure 4) and WAT (309 genes; Figure 4) compared to control diet fed animals. This fits with the lower fat accumulation in HFD-ED animals compared to HFD-corn oil animals, and there was no difference between fat accumulation in HFD-ED and control diet fed groups.

**Figure 5:**
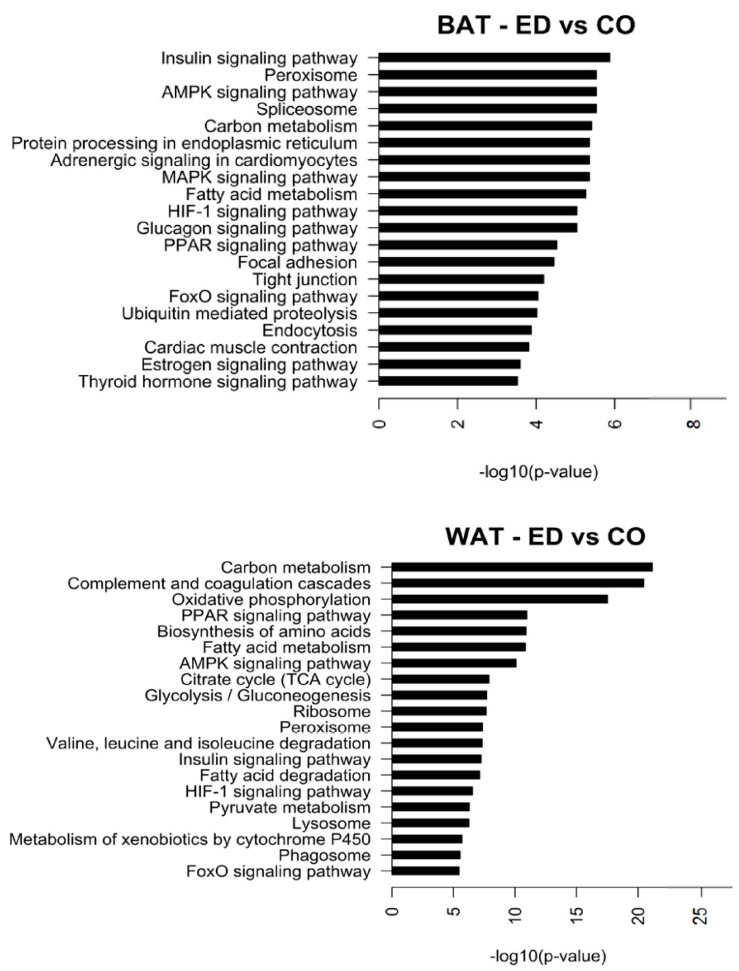
Top 20 pathways regulated in BAT for diet comparison HFD-ED vs. HFD-CO and Top 20 pathways regulated in WAT for diet comparison HFD-ED vs. HFD-CO. Pathway analysis was performed by taking all the up- and down-regulated probes into account.

Based on KEGG pathway analysis, the two different HFD affected in a diverse range of pathways. In BAT, the top regulated pathways involve metabolic activity such as carbon and fatty acid metabolism, hormone signalling, protein degradation and processing (Figure 5). In WAT, the top regulated pathways involve metabolic activities such as oxidative phosphorylation, citric acid cycle, glycolysis/gluconeogenesis. Also, signalling pathways, biosynthesis, and degradation (valine, leucine, and isoleucine) of amino acids (Figure 5). 

### 2.4. GO-Terms Gene-Set Enrichment Analyses of Biological Processes Affected by the HFD Diets in White- and Brown Adipose Tissues

**Figure 6:**
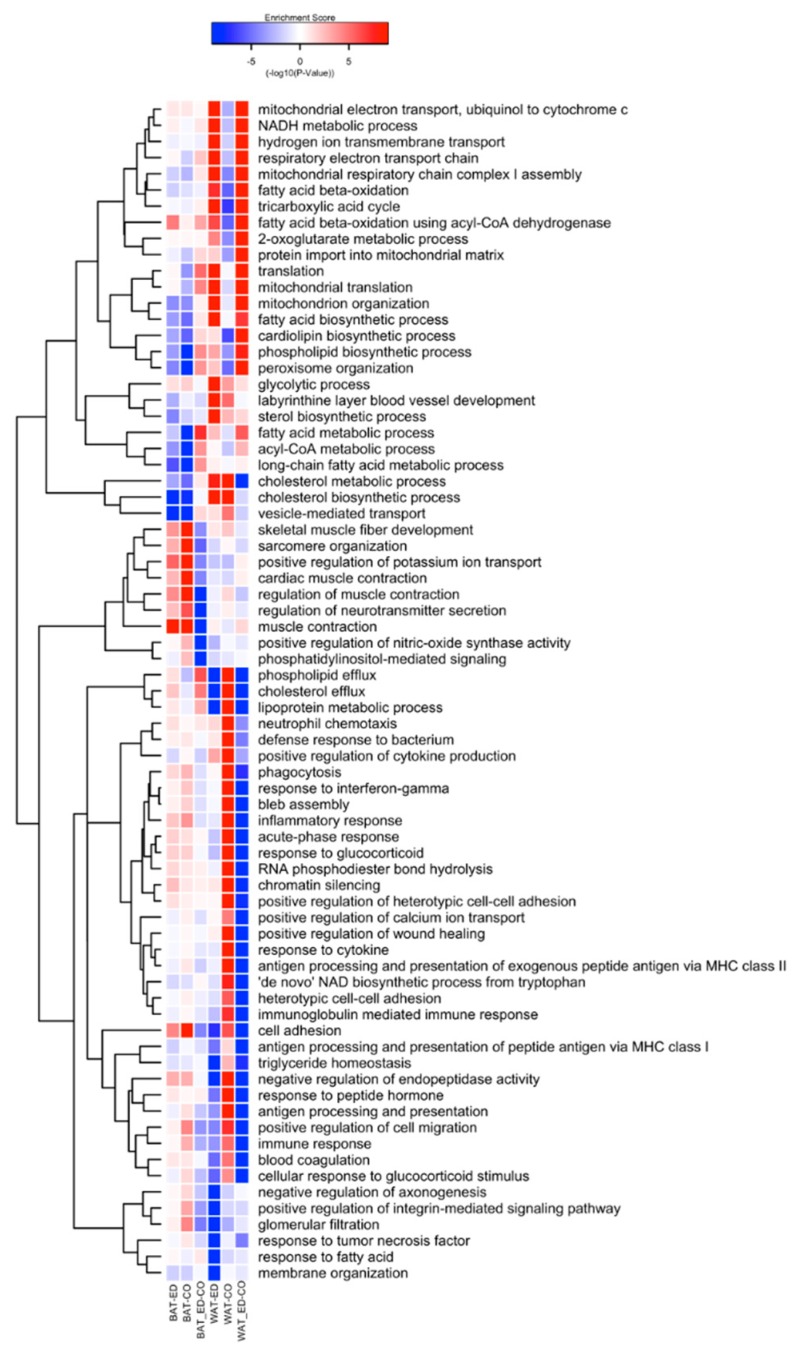
A heatmap showing significant biological process (BP) GO-terms (*p*-value < 10^−10^) for different diet across BAT and WAT after 8 weeks diet intervention. Colour patterns indicate the direction of regulation where red indicates up-regulation and blue indicates down-regulation of immune specific GO-terms. Labels, HFD-ED = HFD-ED vs. control diet; HFD-CO = HFD-corn oil vs. control diet; ED-CO = HFD-ED vs. HFD-corn oil.

In WAT, HFD-ED up-regulated BPs related to mitochondria including electron transport chain, fatty acid β-oxidation, mitochondrial-translation, and organization, whereas, these processes were down-regulated by HFD-corn oil compared with control animals (Figure 6). Furthermore, BPs related to phospholipid- and cholesterol efflux and lipoprotein metabolic processes were strongly down-regulated by HFD-ED but up-regulated by HFD-corn oil compared with control fed mice. Immune related BPs were down-regulated in HFD-ED fed mice including inflammatory response including acute phase, antigen processing and presentation, immunoglobulin-mediated immune response, response to tumor necrosis factor, but were up-regulated in HFD-corn oil fed mice compared with control mice.

One distinct BP, fatty acid beta-oxidation using acyl-CoA dehydrogenase was up-regulated by HFD-ED compared with HFD-corn oil. BPs related to skeletal muscle fibre development, and regulation of muscle contraction were among those up-regulated by both HFDs, and among down-regulated were BPs related to mitochondrial organization, and fatty acid metabolic processes including biosynthesis of fatty acid, phospholipid, cardiolipin, cholesterol and sterol (Figure 6).

### 2.5. Differential Expression of Pparα and Prdm16 in BAT and WAT in Response to HFD-ED 

**Figure 7:**
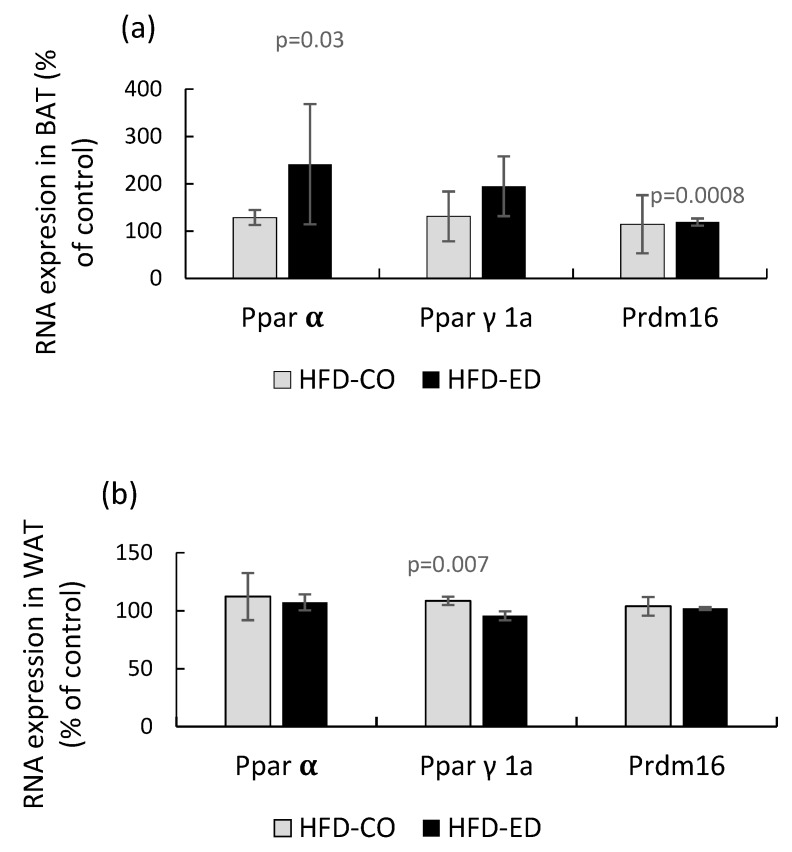
RNA expression of *Pparα*, *Pparγ1a* and *Prdm16* in (**a**) BAT and (**b**) WAT in response to HFD-ED and HFD-CO relative to the control diet. All data for BAT are mean values of 3 tissues (*n* = 3). WAT RNA expression in the HFD-ED group was based on 2 animals due to that the RNA from the third animal did not pass the quality check. Also data on *Prdm16* in the WAT control diet group was based on *n* = 2 (missing value).

The gene expression of *Ppar-α* involved in β-oxidation was significantly higher in response to the HFD-ED compared to the control, such an effect was absent in the HFD-CO group, Figure 7. A small but significant effect of HFD-ED vs. the control diet on the expression of *Prdm16*, involved in thermogenesis, was also observed. No effect of HFD-ED or HFD-CO on *Ppar-α* or *Prdm16* in WAT was detected. The only effect of the HFDs in WAT was a small, but significant, effect of HFD-CO on the gene Ppar γ1a involved in lipogenesis

## 3. Discussion

LC n-3 PUFAs, especially EPA and DHA are proposed to have beneficial effects on human health, that might be explained by their hypolipidemic and anti-inflammatory properties in mammals [23,24]. Undoubtedly, there are a number of factors contributing to the magnitude, and type of effects caused by EPA and DHA supplementation, such as population heterogeneity, dose, background lipid status and diet [18,20]. In this study, male C57Bl/6J mice fed an HFD (i) with 5% corn oil and (ii) 3% corn oil and 2% EPA and DHA enriched oils, similar in total energy led to the differences in weight gain [23] also observed in [25]. EPA and DHA supplementation delayed weight gain, also observed by Miller et al., and were also reflected in lower fat accumulation in BAT and WAT following an 8-week intervention.

Adipose tissues function as specialized energy reservoirs [26] and are capable of expanding to accommodate excess energy in the form of lipids [27]. However, what causes the obesity-induced inflammatory response in adipose tissue is still debated, though it is thought to be related to cellular injury during adipocyte expansion to accommodate a greater volume of triglycerides [28]. Another possible mechanism is that intake of dietary fat up-regulates inflammatory pathways, with saturated fatty acids stimulating the NF-κB pathway [29], possibly via saturated fatty acid binding to Toll-like receptors [30]. Earlier it was found that feeding fish oil, and EPA and DHA individually reduced inflammation in rodent models [28], supporting the likelihood that HFD-ED fed mice were likely to be less exposed to inflammation compared to the mice fed HFD-corn oil.

Determination of fat content and its distribution clearly shows incorporation of EPA and DHA in the phospholipid membrane and lower linoleic and arachidonic acid in the neutral lipid fraction from mice fed HFD-ED, suggesting that not only does EPA and DHA get incorporated into adipocyte membranes, but could also have a downstream effect on production of pro-inflammatory lipid mediators derived from linoleic and arachidonic acid. Lower amounts of arachidonic acid in the phospholipid fraction of adipose tissue in HFD-ED fed mice coupled with down-regulation of genes coding for phospholipase and several members of cytochrome P450 family suggested that the HFD did not stimulate prostaglandin-mediated inflammation in the presence of EPA and DHA to the same extent, as mice fed HFD-corn oil. Nevertheless, increased arachidonic acid levels in tissue phospholipids of HFD-corn oil fed mice, could suggest increase in the arachidonic acid derived eicosanoids such as 2-arachidonoylglycerol and anandamide that are linked with increased food intake, feed efficiency, and obesity development [31]. Another study suggests that the relationship between these fatty acids is bell shaped—that is, at low levels of EPA and DHA, arachidonic acid is closely correlated (concentrations not affected), while at high EPA and DHA levels, there is a negative relationship [32]. Supplementation of EPA and DHA in a high fat diet reduced liver and adipose tissue phospholipid arachidonic acid content [31,33]. This supports our finding that EPA and DHA reduces phospholipid arachidonic acid in adipose tissue, an outcome we also found in liver, skeletal muscle and spleen [23,34,35]. If anti-inflammatory effects can still be observed when arachidonic acid is correlated with EPA and DHA at lower doses, then possibly other mechanisms may play a greater role [23] in stimulating inflammation rather than the change in membrane fatty acid composition per se. The EPA/DHA diet stimulated many pathways related to energy metabolism, and β-oxidation in particular—which also play a role in regulating the inflammatory response [36].

Up-regulation of BPs related to mitochondria and energy metabolism including the electron transport chain, and FA β-oxidation, in mice fed HFD-ED compared with HFD-corn oil point to an important effect on energy metabolism. Analysis of pathway up-regulation supports the up-regulation of fat metabolism, with regulation of the AMP activated kinase pathway which regulates metabolic fuel selection [37], HIF-1 (Hypoxia-inducible factor-1), which regulates oxidative metabolism [38], pyruvate metabolism and FoxO (Forkhead box protein) pathway, which could link EPA and DHA to insulin signaling [39]. An effect on glucose metabolism is further indicated by regulation of the TCA cycle, glycolysis and branched chain amino acid degradation, which all point to effects on glucose metabolism alongside the change to fat metabolism. Follow-up work should include measurements of insulin secretion and sensitivity, especially given the large number of studies that postulate a role for EPA and DHA in improvement of T2D. However, it should be noted that a relationship between LC n-3 PUFA intake from a variety of sources is not conclusively linked to any reduced risk of T2D [40], though the number of clinical trials studying this is limited [41]. The conclusions of the gene array data are strengthened by the differential adipose tissue expression of *Pparα, Pparγ1a*, and *Prdm16* involved in β-oxidation, lipogenesis and thermogenesis, respectively. *Ppar*:s are believed to be primary mediators of the effects of EPA and DHA. *Ppar-α* was increased in BAT of HFD-ED fed mice and Prdm16, in accordance with Pahlavani et. al. [7] who observed increased gene expression in BAT, but not in WAT.

The widespread upregulation of energy metabolism would be expected to result in increased metabolic rate—something that was not measured in the present study. Some clinical trials have found that fish oil supplementation can increase metabolic rate [42], while others have not found any effect [43]. One human study found that while resting metabolic rate was marginally increased by 3 g EPA and DHA/day, there was no effect on FA oxidation [44], which does not support β-oxidation mediated increase in energy expenditure. The wide diversity of results highlights the need for follow-up studies that can specifically address these potential mechanisms, and also the inherent problems of translating mechanisms found in rodent models to outcomes in human intervention trials.

## 4. Materials and Methods 

### 4.1. Animals and Ethical Declaration

Six-week-old male C57BL/6J mice were purchased from the Harlan Laboratories in the Netherlands. They were caged 5–6 per group and acclimatized for three weeks at a local animal facility with 12 h light/dark cycles in Gothenburg, Sweden. They had ad libitum access to study chow (see diet composition for details) and water as previously reported [23]. Two groups were fed a high fat diet to induce obesity, while the third group ate a standard rodent chow and served as a control group. After 8 weeks of dietary intervention, the mice were anaesthetized with > 60 mg/kg intraperitoneal injection of sodium pentobarbital and blood was collected from the heart followed by cervical dislocation. Tissues including WAT and BAT were dissected, weighed and snap-frozen in liquid nitrogen and stored at −80 °C until further use. The study was approved in 2009 by the Animal Ethical Committee at University of Gothenburg, Sweden (Study approval number: 253-2009) [23].

### 4.2. Diet Composition

Mice were fed standard chow (control group) or one of two HFDs that differed only in fatty acids (FAs), whereas other macronutrients were kept the same (Table 2). Briefly, part of the fat component of HFD-corn oil was prepared with 5 % (*w*/*w*) corn oil, while in the HFD-ED, this 5 % was prepared with 2 % (*w*/*w*) EPAX (n-3 enriched) oils (EPA; EPAX 1050 and DHA; EPAX 6015; EPAX AS, Lysaker, Norway) and 3 % (*w*/*w*) corn oil. Hence, in the HFD-ED diet, the corn oil was replaced with 40% EPA and DHA containing triglycerides and 2 % of the diet overall consisted of EPA and DHA. The diet was prepared at Lantmännen AB (Kimstad, Sweden). The feed supply in the cages was changed three times per week during the course of the study.

### 4.3. RNA Isolation, Quality Assurance and Microarray Analysis

White and brown adipose tissues from four different mice were selected for total RNA isolation and microarrays based on the similarity in their body weight, plasma triglyceride, and plasma cholesterol levels (supplementary Table S1). In brief, total RNA was purified using the RNeasy^®^ Plus Universal Mini kit (Qiagen Nordic, Sollentuna, Sweden) following the manufacturer’s instructions. RNA integrity was estimated using RNA 6000 Nano LabChip for Agilent 2100 Bioanalyzer (Agilent Technologies, Santa Clara, California, USA). RNA was quantified by NanoDrop 2000c UV-Vis Spectrophotometer (Thermo Scientific, Wilmington, NC, USA).

Microarray experiments were carried out by the SCIBLU Genomics core facility (Swegene Centre for Integrative Biology at Lund University, Sweden). Briefly, at the facility, the total RNA was labelled and hybridized to MouseWG-6_V2.0 Expression BeadChip (MouseWG-6_V2.0_R3_11278593_A; Illumina, San Diego, CA, USA) containing 45,281 transcripts. The data (raw and normalized) are deposited in SOFT-format at Gene Expression Omnibus (GEO) database under the accession number GSE76622.

Mean intensities from the array fluorescence signals were calculated from illumina bead array files using GenomeStudio Gene Expression software (GSGX v1.9.0). The data was quantile normalized, and variance-stabilizing transformation (VST) was performed using the default setting of *lumiExpresso* function from lumi package, v2.18.0 [45]. The Empirical Bayes method from the Linear Models for Microarray Data (limma) package was then applied to the signals to calculate moderated t- and F-statistics, log odds and differential expression for comparisons between the diets from both adipose tissues [46]. 

A platform for integrative analysis of omics data (Piano) Bioconductor package v.3.0 was used for gene set enrichment analysis (GSEA) for functional inference [47]. Before implementing the gene-set enrichment function from Piano, gene-set collection (gsc) files were prepared for gene ontologies (GO) related to Biological Processes (BPs), Molecular Function (MF) and Cellular Component (CC), and also for GO related to immune system process (GO:0002376). The Piano package was implemented for both global and immune related changes on WAT together with BAT for all diet combinations. In both gene-set analysis, gene-sets with less than 10 and more than 500 genes were excluded. For gene-expression analysis, Benjamini and Hochberg (BH) corrected p-value < 0.001 for at least one diet comparison was considered significant. The Generally Applicable Gene-Set/Pathway Analysis (GAGE) bioconductor package was used for pathway enrichment analysis [48]. Before implementing the gage function, canonical signalling and metabolic pathways from the KEGG mouse database were prepared for better-defined results. The Pathview package, v1.10 was used to visualize the data, in which the pathview function downloads KEGG graph data and renders a pathway map, based on experimental results [49]. The top 20 significantly enriched pathways were plotted as barplots and FDR corrected p-values (q-values) data were transformed to −log10(p_adj_-value).

### 4.4. RT-qPCR Analyses of Selected Genes

#### 4.4.1. Samples

Total RNA in BAT and WAT from three animals (*n* = 3) in the low-fat control, HFD-CO, and the HDF-ED groups were analyzed using a gene expression profiling service (TATAA Biocenter, Gothenburg, Sweden).

#### 4.4.2. Quality Control

The concentration and purity of the samples was measured spectrophotometrically with the Dropsense96 (Unchained Labs, Pleasanton, CA, USA. The RNA integrity was analyzed using capillary gel electrophoresis on the Fragment Analyzer (Agilent, Santa Clara, CA, USA) and the Standard Sensitivity RNA kit (Agilent, Cat No. DNF-471-0500). All RNA samples except for one sample from WAT in the HDF-ED group, passed the quality check. The data originating from the faulty sample were excluded in the statistical analyses.

#### 4.4.3. Normalization and cDNA Synthesis 

The RNA samples were reverse transcribed using the GrandScript cDNA synthesis kit (TATAA Biocenter, Cat No. A103b, TATAA Biocenter AB, Gothenburg, Sweden according to manufacturer’s instructions in single 20 μL reactions. Prior to the reverse transcription all samples where normalized to 200 ng/μL; giving a total input of 1000 ng RNA into the reverse transcription reaction. 5 μL of samples with concentrations lower than 200 ng/μL where loaded directly without normalizations. Water was used as RT-NTC and included in the analysis. The cDNA samples were diluted 10 times in RNase free water and analyzed on all 12 assays in the TATAA Mouse Reference Gene Panel (TATAA Biocenter, Cat No. A102P).

#### 4.4.4. cDNA Synthesis and qPCR 

Mouse ValidPrime assay (TATAA Biocenter, Cat No. A106P25) was used to compensate for possible gDNA contamination and mouse gDNA (Bioline, Cat No. BIO-35027) was included in the analysis. NTCs were included as a control for contamination of reagents and all samples were run in duplicates. The master mixes were prepared with TATAA Probe Low ROX GrandMaster Mix (TATAA Biocenter, Cat No. TA02-625LR). All pipetting was performed by a pipetting robot (EpMotion 5070, Eppendorf, Hamburg, Germany. The qPCR was run on the Quantstudio 12K Flex platform (ThermoFisher, Waltham, MA, USA. The Cq values were determined using the relative thresholding method in the Quantstudio 12K Flex Software (ThermoFisher, Waltham, MA, USA, after which GenEx (MultiD) version 7.0.2.164 was used to perform data pre-processing including ValdPrime correction. The two most suitable reference genes were evaluated using the NormFinder and GeNorm algorithms. The final selection of the two best reference genes was made using NormFinder. 

#### 4.4.5. Gene Expression Profiling 

Three Bio-Rad assays targeting Genes of interest GOIs (Table 3), two reference gene assays and ValidPrime (TATAA Biocenter, Cat No. A106P25) were used for expression profiling of the mouse samples. cDNA was diluted 3 times before qPCR. RNase free water was used as an NTC and included along with RT-NTC and mouse gDNA (Bioline, Cat No. BIO-35027, London, UK in the analysis. The master mixes were prepared with TATAA Probe Low ROX GrandMaster Mix (TATAA Biocenter, Cat No. TA02-625LR). All pipetting was performed by a pipetting robot (EpMotion 5070, Eppendorf). The qPCR was run on the Quantstudio 12K Flex platform (ThermoFisher). The Cq values were determined using the relative threshold method in the Quantstudio 12K Flex Software (ThermoFisher), after which GenEx (MultiD) was used to perform data preprocessing and to calculate the data Cq for the GOIs relative to reference genes. 

### 4.5. Fatty Acid Analysis by Gas-Chromatography Mass Spectrometry

Approximately, 100 mg WAT and BAT tissues were weighed and freeze-dried for FA extraction using the Folch total lipid extraction method [50]. Neutral lipids, free fatty acids, and phospholipid fractions were separated, and each fatty acid fraction was quantified using gas chromatography mass spectrometry (GC-MS), as previously described [23]. Fatty acids were quantified against internal phospholipid (C17:0) and triglyceride (C19:0) standards (Nu-Chek Prep, Inc., Elysian, MN, USA) and was expressed as relative percentage of total lipid fractions. The fatty acid methyl esters (FAME) were separated by gas chromatography (Agilent 7890A, Santa Clara, CA) and detected with mass spectroscopy. Chemstation software (Agilent Technologies, Santa Clara, CA) was used for evaluation. The samples were separated on a VF-WAX (30 × 0.25 × 0.25 μm d_F_) column (Agilent Technologies) and detected with a 5975C inert mass spectrometer (Agilent Technologies).

### 4.6. Statistical Analysis

Physiological data were considered different for *p*-value < 0.05 based on one-way ANOVA with post-hoc Tukey-Honest significant difference test (Tukey-HSD). For the microarray data and GSEA, BH adjusted *p*-value < 0.001 was considered significant and used as a cut-off value for considering data for biological interpretation. The data are presented as mean ± SEM. Statistical and microarray data were analysed in the R Studio environment (Version 0.99.903—© 2009-2016 RStudio, Inc.) using Bioconductor packages.

## 5. Conclusions

HFD-ED fed mice showed reduced lipid accumulation and levels of the pro-inflammatory fatty acid arachidonic acid in both white and brown adipose tissues, compared with HFD-corn oil fed animals. The transcriptomic analysis showed changes in β-oxidation pathways, supporting the decreased lipid accumulation in the HFD-ED fed mice. Therefore, our data suggests that EPA and DHA supplementation of a high fat diet may be anti-inflammatory, as well as reduce lipid accumulation in adipose tissues. 

## Figures and Tables

**Table 1:** Changes in body weight of the mice fed control (*n* = 9), high fat diet—EPA and DHA (HFD-ED) (*n* = 12), or high fat diet—corn oil (HFD-corn oil) (*n* = 12). Letters a and b denotes significant difference *p* ≤ 0.05

Parameter	Control	HFD-ED	HFD-Corn Oil
Initial body weight (g)	27.50 ± 0.80	28.60 ± 0.80	24.30 ± 0.50
Final body weight (g)	31.40 ± 1.00	33.80 ± 0.80	36.20 ± 0.90
Change in body weight (g)	3.90 ± 0.50 ^a^	5.20 ± 0.30 ^a^	8.60 ± 0.50 ^b^

**Table 2:** Composition of control, high fat diet— eicosapentaenoic acid (EPA) and docosahexaenoic acid (DHA) (high fat diet (HFD)-ED), or high fat diet—corn oil (HFD-CO) diet [23].

Ingredient (g/100 g Diet)		Control	HFD-ED	HFD-CO
Protein	Casein	22.20	25.60	25.60
Carbohydrates	Sucrose	5.00	10.00	10.00
	Corn starch	56.00	34.80	34.80
	Cellulose	5.00	5.80	5.80
Fat	Total	5.00	15.00	15.00
	Corn oil	2.50	3.00	5.00
	Coconut oil	2.50	10.00	10.00
	EPAX oils ^a^	0.00	2.00	0.00
Minerals ^b^		2.00	2.50	2.50
Micro nutrients ^c^		3.00	3.00	3.00
Choline bitartrate		1.60	2.00	2.00
Cholesterol		0.00	1.00	1.00
Methionine		0.20	0.30	0.30
Energy content (kJ/100g)		1599	1752	1752
	Protein E%	24	25	25
	Carbohydrate E%	65	44	44
	Fat E%	12	32	32
Fatty acid composition ^d^ (mg/g diet)				
	C10:0	0.20	1.47	1.33
	C12:0	2.37	7.58	7.72
	C14:0	1.54	4.58	4.78
	C16:0	1.90	3.44	3.59
	C18:0	0.68	2.26	2.49
	SFA	6.70	19.33	19.91
	C18:1 n-9	2.82	4.80	5.26
	MUFA	2.82	4.80	5.26
	C18:2 n-6	3.62	5.03	7.36
	C18:3 n-6	0.12	0.22	0.26
	Total n-6 PUFA	3.74	5.26	7.62
	C20:5 n-3 (EPA)	0.00	2.03	0.01
	C22:6 n-3 (DHA)	0.00	4.58	0.01
	Total n-3 PUFA	0.00	6.61	0.02

^a^ EPAX 1050. EPAX 6015; ^b^ CaCO_3_ (57.7%); KCl (19.9%); KH_2_PO_4_ (11.9%); MgSO_4_ (10.4%); ^c^ Corn starch (98.22%); Ca(IO_3_)_2_ (0.0007%); CoCO_3_ (0.064%); CuO (0.02%); FeSO_4_ (0.5%); MnO_2_ (0.035%); Na_2_MoO_4_ (0.001%); NaSeO_3_ (0.0007%); ZnO (0.1%); Vitamin A (0.013%); B_2_ (Riboflavin-5-phosphate sodium; 0.027%); B3 (0.1%); B_5_ (Ca Pantothenate; 0.057%); B_6_ (0.023%); B_7_ (0.0007%); B_9_ (0.007%); B_12_ (0.00008%); D_3_ (0.007%); E (0.25%); K (0.003%); ^d^ Diet analyses of fatty acids were performed in triplicates, and the data was obtained by gas chromatography mass spectroscopy.

**Table 3:** Bio-Rad assays targeting Genes of interest (GOIs).

Target Gene	Assay ID	Probe Dye	Comment
*Pparα*	qMmuCEP0054952	FAM	GOI
*Pparγ1a*	qMmuCIP0030057	FAM	GOI
*Prdm16*	qMmuCEP0057138	FAM	GOI

## Supplementary Materials

Supplementary materials can be found at https://www.mdpi.com/1422-0067/20/23/5895/s1.
