# Supplementary material for: The Omega-3 Fatty Acids EPA and DHA, as a Part of a Murine High-Fat Diet, Reduced Lipid Accumulation in Brown and White Adipose Tissues
Source: Int J Mol Sci. 2019 Nov 24;20(23):5895. doi: 10.3390/ijms20235895 (PMC6928976; doi:10.3390/ijms20235895)
Supplement: Supplementary file 1 [file ijms-20-05895-s001.pdf]

Supplemental Table S1 Selection of animals for microarray analysis.

Animals group	Animal ID	Body weight (g)	plasma TG (mM)	plasma chol. (mM)
Control	3527	31.2	1.24	8.2
Control	7343	37.4	0.92	4.8
**Control**	5122	29.8	1.12	6,0
**Control**	7037	30,0	0.52	4.6
**Control**	3377	28.1	1,00	4.8
**Control**	0311	28.4	0.88	6.4
Control	4356	33,0	1.12	5.2
Control	5807	32.8	0.72	5.2
Control	0104	31.6	0.4	4.8
**HFD_EPA/DHA**	2824	30.5	0.92	5.4
**HFD_EPA/DHA**	0864	26.7	1.12	7,0
HFD_EPA/DHA	6534	30	0.84	6,0
HFD_EPA/DHA	7887	30.3	0.68	4.8
HFD_EPA/DHA	6304	30.2	1.04	5.4
**HFD_EPA/DHA**	7011	23.5	0.92	6.4
HFD_EPA/DHA	5345	31.1	1.2	4.2
**HFD_EPA/DHA**	6572	29.4	2,00	4.6
HFD_EPA/DHA	2790	27.1	1.16	6,0
HFD_EPA/DHA	5304	32	1.16	5.2
HFD_EPA/DHA	7538	27.4	1.16	8,0
HFD_EPA/DHA	4869	24.7	1,00	2.8
**HFD_corn oil**	1787	26	0.64	4
HFD_corn oil	9895	25.2	0.48	3.2
**HFD_corn oil**	6524	21.3	0.56	3.6
HFD_corn oil	5592	24.3	0.44	3
HFD_corn oil	5008	24.8	0.56	5
**HFD_corn oil**	3014	24.4	0.84	6.4
HFD_corn oil	6523	25.9	0.72	5.6
HFD_corn oil	9101	25	0.64	5.4
**HFD_corn oil**	3358	27	0.92	6
HFD_corn oil	2539	23.5	0.72	7
HFD_corn oil	1091	22.2	1.12	10
HFD_corn oil	1088	22.2	0.4	3.6

The animals marked in bold letters were selected for microarray analysis.
